# Revealing Cell‐Free Mitochondrial DNA Breakage Patterns as Novel Biomarkers for Sepsis

**DOI:** 10.1002/advs.202414159

**Published:** 2025-07-26

**Authors:** Haitang Liao, Ruiyan Ma, Shuai Hao, Xuxin Tan, Xue Zeng, Rui Song, Bing Chen, Zhezhe Cao, Wei Shen, Zhenchun Luo, Jianfeng Huang, He Huang, Liangming Liu, Chenyang Duan

**Affiliations:** ^1^ Pinnacle Disciplinary Group (Department of Anesthesiology) The Second Affiliated Hospital of Chongqing Medical University Chongqing 400010 P. R. China; ^2^ Department of Infectious Diseases Key Laboratory of Molecular Biology for Infectious Diseases (Ministry of Education) Institute for Viral Hepatitis the Second Affiliated Hospital of Chongqing Medical University Chongqing 400010 P. R. China; ^3^ Department of Intensive Care Unit Chongqing Hospital of Traditional Chinese Medicine Chongqing 400011 P. R. China; ^4^ Department of Cardiovascular Surgery Xinqiao Hospital Army Medical University Chongqing 400037 P. R. China; ^5^ Research Institute of General Surgery Jinling Hospital Affiliated Hospital of Medical School Nanjing University Nanjing 210000 P. R. China; ^6^ Sinotech Genomics Co., Ltd Shanghai 201103 P. R. China; ^7^ State Key Laboratory of Trauma and Chemical Poisoning Department of Shock and Transfusion Army Medical Center Army Medical University Chongqing 400042 P. R. China

**Keywords:** cell‐free mitochondrial DNA, diagnosis, DNA fragmentomics, prognosis, sepsis biomarker

## Abstract

Accurate early diagnosis and prognosis of sepsis remain major clinical challenges. This study explores specific plasma cell‐free mitochondrial DNA (cfmtDNA) breakage patterns as potential biomarkers for sepsis. Plasma samples from ten non‐sepsis control patients and 63 sepsis patients are analyzed using mitochondrial DNA fragmentomics, revealing distinct breakage sites in the RNR2 (positions 2474–2478) and COX2 (positions 7761, 7775, 7776, 7777, and 7783) regions, which are intact in healthy individuals but exhibited high‐frequency breakage in sepsis patients. Diagnostic models based on these breakage sites show superior accuracy for sepsis detection (AUC = 0.865) and prognosis prediction (AUC = 0.809) compared to traditional cfmtDNA copy number assessments. Notably, COX2 breakage frequency correlated with inflammatory markers and SOFA scores, highlighting its prognostic potential. Mechanistic analyses suggest that reduced protein binding in sepsis may increase cfmtDNA susceptibility to cleavage by bacterial restriction endonucleases. These findings indicate that plasma cfmtDNA breakage characteristics can serve as valuable biomarkers for early sepsis detection and therapeutic monitoring.

## Graphical abstract

Uncovering distinct plasma cfmtDNA breakage patterns opens new frontiers for early sepsis detection and prognosis. Unique cleavage hotspots in RNR2 and COX2 regions outperform traditional biomarkers, while mechanistic insights suggest bacterial endonuclease involvement. This study reveals a previously unrecognized layer of mitochondrial DNA vulnerability, offering exciting opportunities for precision diagnostics and therapeutic monitoring in sepsis.

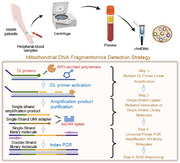

## 1 Introduction

Sepsis is a life‐threatening organ dysfunction caused by dysregulated host responses to infection, not the infection itself.^[^ ^1^ ^]^ It is a leading cause of death among critically ill patients in the perioperative period. Globally, ≈49 million people develop sepsis annually, with 11 million sepsis‐related deaths accounting for nearly 20% of all deaths worldwide.^[^ ^2^ ^]^ Timely diagnosis and early management are crucial to improve patient outcomes and focus on early recognition, prompt and appropriate antibiotic therapy, hemodynamic support, and infection source control. However, the current diagnostic criteria for sepsis rely on clinical presentation and laboratory findings, which are often ambiguous. Traditionally, blood infections are identified through culture, quantitative PCR (qPCR), or by measuring inflammatory markers such as C‐reactive protein (CRP) and procalcitonin. These methods are limited by long turnaround times, narrow pathogen spectra, and poor specificity. For example, the blood culture positivity rate in hospitalized patients with community‐acquired sepsis is only 14%. Additionally, commonly used markers of myocardial injury, such as cTnT and cTnI, do not begin to increase until at least six hours after symptom onset, with low sensitivity in the early stages of septic cardiomyopathy.^[^ ^3^ ^]^ Consequently, the sequential organ failure assessment (SOFA) score—a key diagnostic indicator of sepsis—is based on these delayed and low‐sensitivity biochemical markers, making early recognition and diagnosis particularly challenging.

Mitochondria contain their own genetic information and are pivotal in innate immune responses and energy production. For example, in pulmonary sepsis caused by severe acute respiratory syndrome coronavirus 2 (SARS‐CoV‐2), mitochondria serve as frontline defenders against viral invasion.^[^ ^4^ ^]^ Meanwhile, SARS‐CoV‐2 can exploit mitochondrial protein translocation mechanisms to localize its encoded proteins to the mitochondria, disrupting normal host cell functions.^[^ ^5^ ^]^ Moreover, mitochondria are highly sensitive to the inflammatory and hypoxic damage that occurs during sepsis, with changes in mitochondrial quality within vital organs often preceding tissue damage detectable by traditional blood biochemical tests and imaging. Hence, monitoring mitochondria‐related indicators for the early diagnosis of sepsis may prove effective, providing a new perspective for detecting cellular damage before it manifests as conventional clinical indicators. Although recent studies have focused on the role of mitochondria‐related indicators in improving the diagnostic efficacy of sepsis, most have concentrated on identifying significantly differentially expressed mitochondrial proteins as potential biomarkers.^[^ ^6^, ^7^ ^]^ While these studies have made progress in enhancing diagnostic performance, the sensitivity and stability of these mitochondrial biomarkers are not well established, and their clinical application faces several challenges.

Circulating cell‐free mitochondrial DNA (cfmtDNA) has gained increasing attention as a potential clinical biomarker.^[^ ^8^ ^]^ This DNA is found in the bloodstream without a surrounding cell membrane and typically originates from the mitochondria of damaged tissue cells. It offers significant advantages, including high abundance, low detection cost, and strong disease correlation. First, compared with the two copies of nuclear DNA (nDNA), each cell contains hundreds of copies of mitochondrial DNA (mtDNA), making cfmtDNA highly suitable for early disease screening. Additionally, mtDNA is only 16 kb in length, less than one‐millionth the size of nDNA, making full‐length sequencing highly cost‐effective and enhancing its feasibility for widespread screening. Furthermore, the immunogenic properties of mtDNA suggest that it plays a crucial role in host immune response. When cells are damaged or stressed, mtDNA is released into the bloodstream, where it acts as a target for pattern recognition receptors (such as Toll‐like receptor (TLR)‐9), activating immune cells through the NLRP3 inflammasome, cGAS–STING pathway, etc., inducing inflammatory cytokine production, and triggering an inflammatory response.^[^ ^9^ ^]^

Blood cfmtDNA copy numbers are significantly elevated in patients with acute respiratory distress syndrome (ARDS) and sepsis and are strongly associated with 28‐day survival rates.^[^ ^10^ ^]^ Additionally, among patients admitted to the intensive care unit (ICU) with septic shock, those with elevated plasma cfmtDNA levels (particularly ND6) at admission are more likely to develop secondary infections and experience significantly increased 90‐day mortality rates.^[^ ^11^ ^]^ While these studies underscore the importance of cfmtDNA in assessing the risk of sepsis and predicting mortality, they also highlight the limitations of traditional cfmtDNA copy number analysis. First, as mtDNA lacks nucleosome protection, it is susceptible to degradation by nucleases in the blood, resulting in ∼50 bp fragments, which are not readily detected by current analysis techniques.^[^ ^12^ ^]^ Second, the numerous variations and breakage sites in mtDNA indicate that conventional PCR with dual primers may encounter primer‐binding regions affected by mutations or breakages, leading to amplification bias or reduced efficiency in identifying and quantifying low‐frequency mtDNA variants in the blood. Therefore, other mtDNA characteristics should be explored to identify more suitable biomarkers for diagnosing and prognosis sepsis and other severe acute conditions.

In this study, we applied single‐primer amplification and library construction techniques to enhance the detection of ultrashort circulating mtDNA fragments.^[^ ^13^ ^]^ We further utilized mitochondrial DNA fragmentomics to explore their potential in the early diagnosis and prognosis of clinical sepsis. Our central hypothesis is that specific plasma cf‐mtDNA breakage patterns reflect underlying mitochondrial stress responses during sepsis and may serve as novel biomarkers for both its diagnosis and prognosis. By identifying these characteristic breakage sites in the plasma cfmtDNA of septic patients, we aim to provide a more specific and sensitive approach for detecting sepsis‐related cf‐mtDNA signatures, ultimately improving the early identification and clinical management of sepsis in patients.

## 2 Results

### 2.1 Plasma cfmtDNA Breakage Patterns are More Specific Indicators of Sepsis Onset than Increased mtDNA Copy Number

Ten non‐sepsis control patients (NC group) and 63 patients diagnosed with sepsis were included in the study. Their basic demographic and clinical information is summarized in **Table** **1**. Among the patients with sepsis, 31 had sepsis due to pulmonary infections such as COVID‐19 (PS group), and 32 had sepsis caused by abdominal infections such as gastrointestinal perforations (AS group). Of the 31 admitted patients with PS, 19 (61.3%) had fatal outcomes (PSD subgroup), while 12 (38.7%) recovered (PSR subgroup). Similarly, of the 32 patients with AS, 17 (53.1%) had fatal outcomes (ASD subgroup), and 15 (46.9%) recovered (ASR subgroup).

**Table 1:** Basic Clinical characteristics of included patients.

	NC group	PS group		P value (PSD vs PSR)	AS group		P value (ASD vs ASR)	P value (PS vs AS)
		PSD	PSR		ASD	ASR		
Number	10	19	12		17	15		
Male (%)	5 (50.0%)	15 (78.9%)	5 (41.7%)	0.056	13 (76.5%)	12 (80.0%)	1.000	0.093
Age (years)	57.60 ± 15.63	69.63 ± 13.95	66.58 ± 16.19	0.595	69.12 ± 14.33	59.73 ± 12.89	0.062	0.203
Laboratory data								
Mean arterial pressure (mmHg)	119.80 ± 13.21	92.42 ± 18.07	91.42 ± 12.56	0.868	102.18 ± 22.27	103.80 ± 10.93	0.800	0.101
White blood cell count (10^9/L)	5.78 ± 1.28	11.75 ± 4.65	8.21 ± 4.03	0.012	10.61 ± 3.32	8.40 ± 4.36	0.034	0.875
Neutrophil percentage (%)	59.03 ± 8.37	93.74 ± 7.21	83.93 ± 11.19	0.001	88.03 ± 8.02	85.40 ± 7.54	0.349	0.135
Haemoglobin (g/L)	134.80 ± 15.48	103.79 ± 18.59	100.67 ± 21.47	0.671	91.29 ± 26.69	100.80 ± 21.98	0.284	0.23
Hematocrit (%)	40.79 ± 6.11	28.14 ± 6.22	28.45 ± 7.95	0.902	28.05 ± 8.54	30.45 ± 6.55	0.386	0.62
SOFA score at admission	0	7.58 ± 4.36	5.25 ± 3.60	0.133	9.59 ± 3.59	6.93 ± 4.20	0.063	0.113
CRP (mg/L) at admission	9.74 ± 8.50	107.49 ± 109.19	60.18 ± 53.07	0.118	136.17 ± 82.97	87.78 ± 54.59	0.064	0.257
PCT (ug/L) at admission	0.12 ± 0.14	1.88 ± 2.93	2.05 ± 3.85	0.890	34.39 ± 44.03	10.95 ± 27.23	0.085	0.003

SOFA‐ sequential organ failure assessment; CRP‐ C‐reactive protein; PCT‐ Procalcitonin; NC group‐ non‐sepsis control patients; PS group‐ sepsis patients caused by pulmonary infection; PSD subgroup‐ PS death group; PSR subgroup‐ PS recovery group; AS group‐ sepsis patients caused by abdominal infection; ASD subgroup‐ AS death group; ASR subgroup‐ AS recovery group.

**Figure 1:**
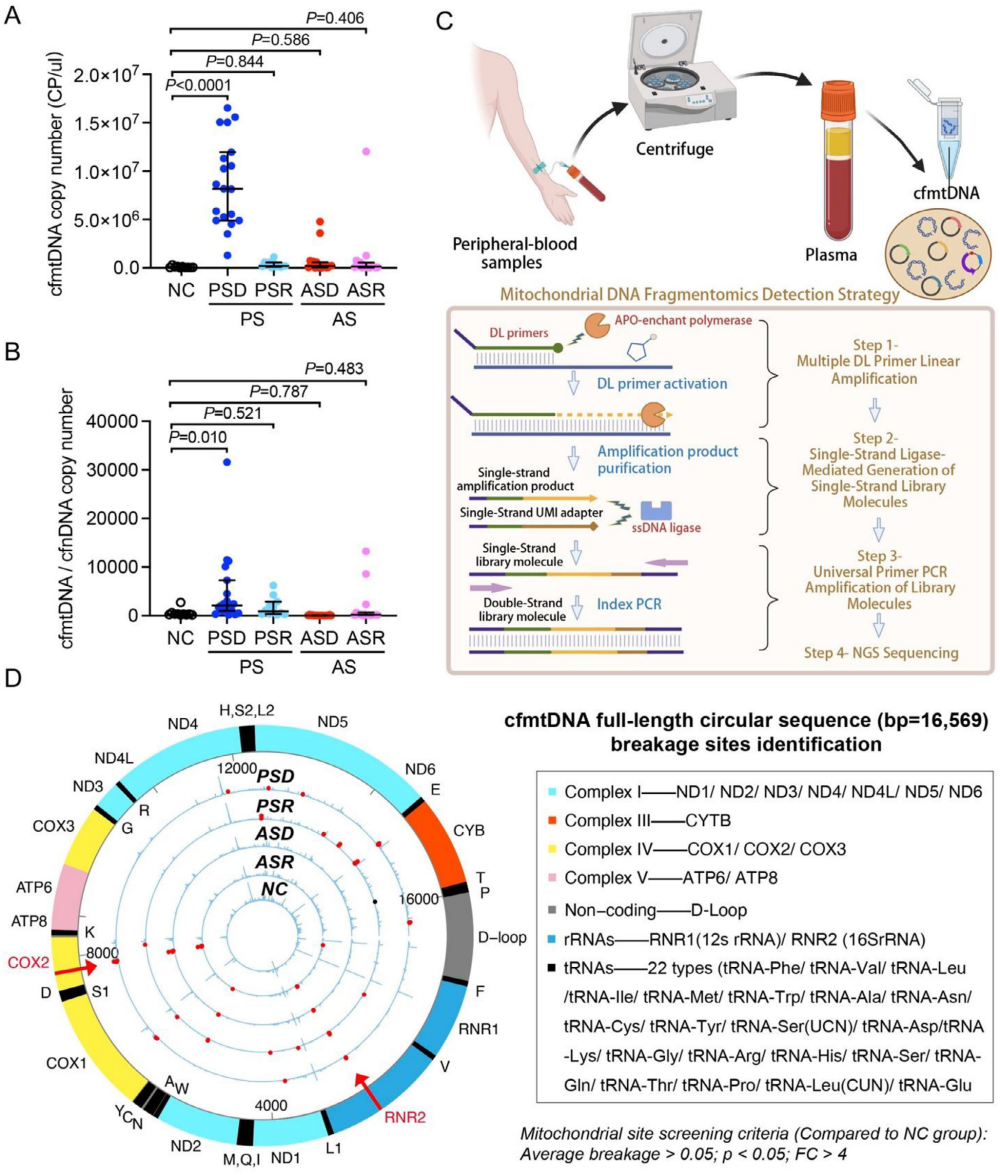
Plasma cfmtDNA copy number and fragmentation patterns in patients with sepsis. A) Comparison of plasma cfmtDNA copy number across different sepsis subgroups. B) Normalized cfmtDNA/cfnDNA copy number across different sepsis subgroups. C) Mitochondrial DNA fragmentomics detection strategy based on single‐primer amplification and library construction technique. D) Identification of cfmtDNA breakage sites in the full‐length circular mtDNA sequence (bp = 16569). NC group: non‐sepsis control patients (n = 10); PS group: patients with sepsis caused by pulmonary infection (n = 31); PSD subgroup: PS death group (n = 19); PSR subgroup: PS recovery group (n = 12); AS group: patients with sepsis caused by abdominal infection (n = 32); ASD subgroup: AS death group (n = 17); ASR subgroup: AS recovery group (n = 15). *p* < 0.05 was considered statistically significant.

Plasma samples were collected from patients upon admission, and plasma cfmtDNA copy numbers were assessed. Only the PSD subgroup exhibited significantly higher plasma cfmtDNA copy numbers at admission than the NC group (P < 0.05), whereas the PSR, ASD, and ASR subgroups showed no significant differences compared with the NC group (P > 0.05; **Figure** **1**A). To minimize the potential impact of nonspecific amplification or competitive inhibition by cell‐free nuclear DNA (cfnDNA) on the cfmtDNA copy number, the cfmtDNA/cfnDNA ratio was normalized; the resulting trends were consistent (Figure 1B). These findings suggest that changes in plasma cfmtDNA copy numbers at admission are not ideal early indicators of sepsis and are primarily indicative of clinical outcomes in patients with severe pulmonary infections leading to sepsis.

To explore how plasma cfmtDNA specifically reflects the characteristics of mtDNA release during sepsis, we not only focused on the overall changes in cfmtDNA quantity but also conducted an in‐depth analysis of the gene fragment characteristics of cfmtDNA released into the plasma during sepsis (Figure 1C; Table S1, Supporting Information). Mitochondrial DNA fragmentation revealed that nearly all patients with sepsis exhibited consistent breakage at specific sites in the plasma cfmtDNA collected at admission, particularly at five consecutive sites in the RNR2 region (positions 2474, 2475, 2476, 2477, and 2478) and five relatively consecutive sites in the COX2 region (positions 7761, 7775, 7776, 7777, and 7783) (Figure 1D). These results suggest that, compared to cfmtDNA copy numbers, the fragmentation patterns of cfmtDNA gene segments in the plasma at admission may serve as more effective biomarkers for sepsis.

### 2.2 High‐Frequency Breakage in the RNR2 and COX2 Regions of Plasma cfmtDNA at Admission can be used for Early Diagnosis of Sepsis

**Figure 2:**
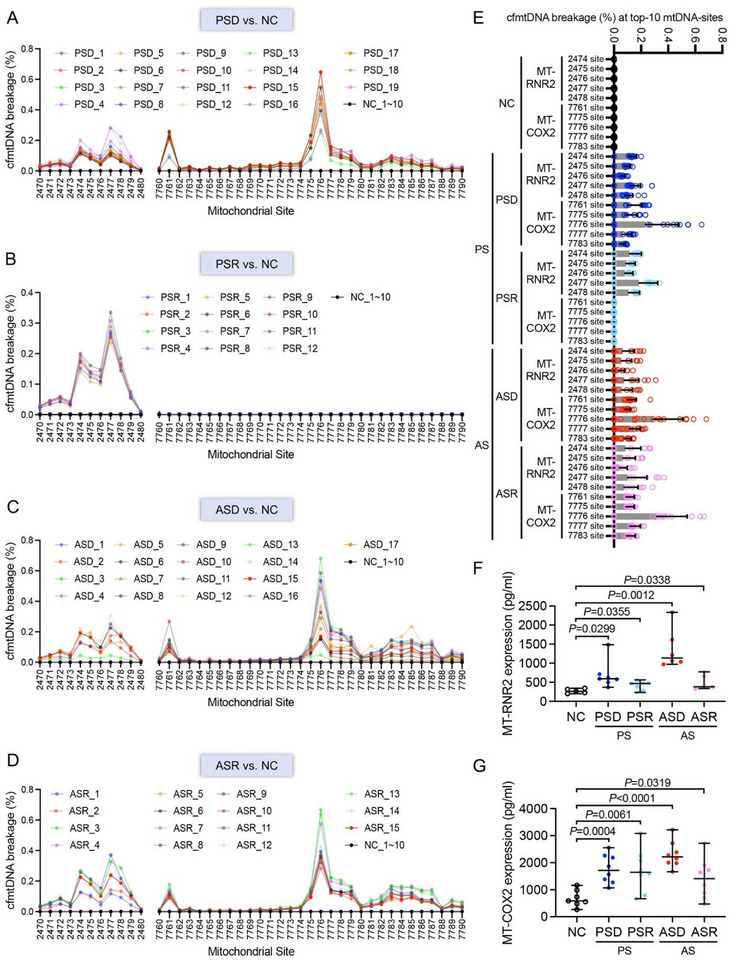
Plasma cfmtDNA breakage frequency (%) at top ten breakage sites in patients with sepsis. Linear sequence charts of cfmtDNA breakage frequency (%) for each individual in the PSD A), PSR B), ASD C), and ASR D) groups. E) cfmtDNA breakage frequency at the top ten breakage sites across the sepsis subgroups. Plasma F) MT‐RNR2 and G) MT‐COX2 levels across different sepsis subgroups determined via ELISA. NC group: non‐sepsis control patients (n = 10); PS group: patients with sepsis caused by pulmonary infection (n = 31); PSD subgroup: PS death group (n = 19); PSR subgroup: PS recovery group (n = 12); AS group: patients with sepsis caused by abdominal infection (n = 32); ASD subgroup: AS death group (n = 17); ASR subgroup: AS recovery group (n = 15). *p* < 0.05 was considered statistically significant.

We conducted a detailed analysis of ten specific breakage sites within the plasma cfmtDNA sequences of all enrolled patients. These ten cfmtDNA breakage sites were entirely conserved in the NC group, with none of the ten plasma samples from non‐sepsis control patients exhibiting breakages at these locations. In contrast, varying degrees of breakage were observed in plasma samples collected at admission from 63 patients with sepsis across the PSD, PSR, ASD, and ASR groups (**Figure** **2**A–D). The breakage frequency of these ten cfmtDNA sites across different groups indicated that the specific cleavage of circulating cfmtDNA in the RNR2 and COX2 regions serves as a highly specific marker for sepsis onset (Figure 2E). To further elucidate the impact of these specific breakages, we examined the fragmentation patterns in the RNR2 and COX2 regions of plasma cfmtDNA. ELISA results demonstrated a significant increase in fragments in the RNR2 and COX2 regions in the PSD, PSR, ASD, and ASR groups compared with the NC group (*p* < 0.05; Figure 2F,G). Hence, detecting specific breakages in plasma cfmtDNA through mitochondrial DNA fragmentomics offers markedly improved sensitivity for sepsis detection compared with traditional cfmtDNA copy number analysis.

**Figure 3:**
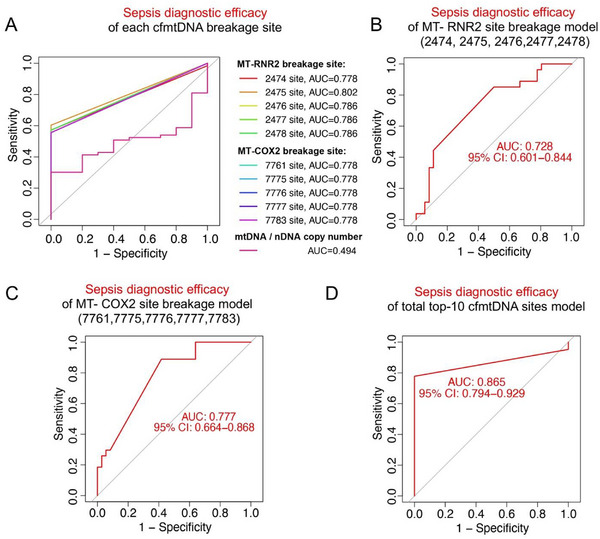
Sepsis diagnostic efficacy of plasma cfmtDNA fragmentation patterns. A) Sepsis diagnostic efficacy of each cfmtDNA breakage site compared with mtDNA/nDNA copy number. B) Sepsis diagnostic efficacy of MT‐RNR2 breakage site model, including sites 2474, 2475, 2476, 2477, 2478. C) Sepsis diagnostic efficacy of MT‐COX2 breakage site model, including sites 7761, 7775, 7776, 7777, and 7783. D) Sepsis diagnostic efficacy of total top‐10 cfmtDNA sites model.

We also evaluated the diagnostic efficacy of plasma cfmtDNA breakage patterns in sepsis. ROC curve analysis revealed that, when used as diagnostic markers, individual cfmtDNA breakage sites exhibited area under the curve (AUC) values ranging from 0.778 to 0.802, significantly surpassing the diagnostic performance of the cfmtDNA copy number (mtDNA/nDNA copy ratio), with an AUC of 0.494 (**Figure** **3**A). When the five consecutive breakage sites in the RNR2 region or COX2 region were utilized as composite diagnostic models for sepsis, the diagnostic performance reached AUC values of 0.728 (Figure 3B) and 0.777 (Figure 3C), respectively. When all ten cfmtDNA breakage sites were combined into an overall diagnostic model, the performance improved to an AUC of 0.865 (Figure 3D). These findings confirm that specific breakages in the RNR2 and COX2 regions of plasma cfmtDNA provide significantly higher sensitivity and specificity for sepsis diagnosis than traditional cfmtDNA copy number analysis.

### 2.3 High‐Frequency Breakage Severity in the COX2 Region of Plasma cfmtDNA at Admission can Serve as an Early Indicator of Poor Prognosis in Sepsis

**Figure 4:**
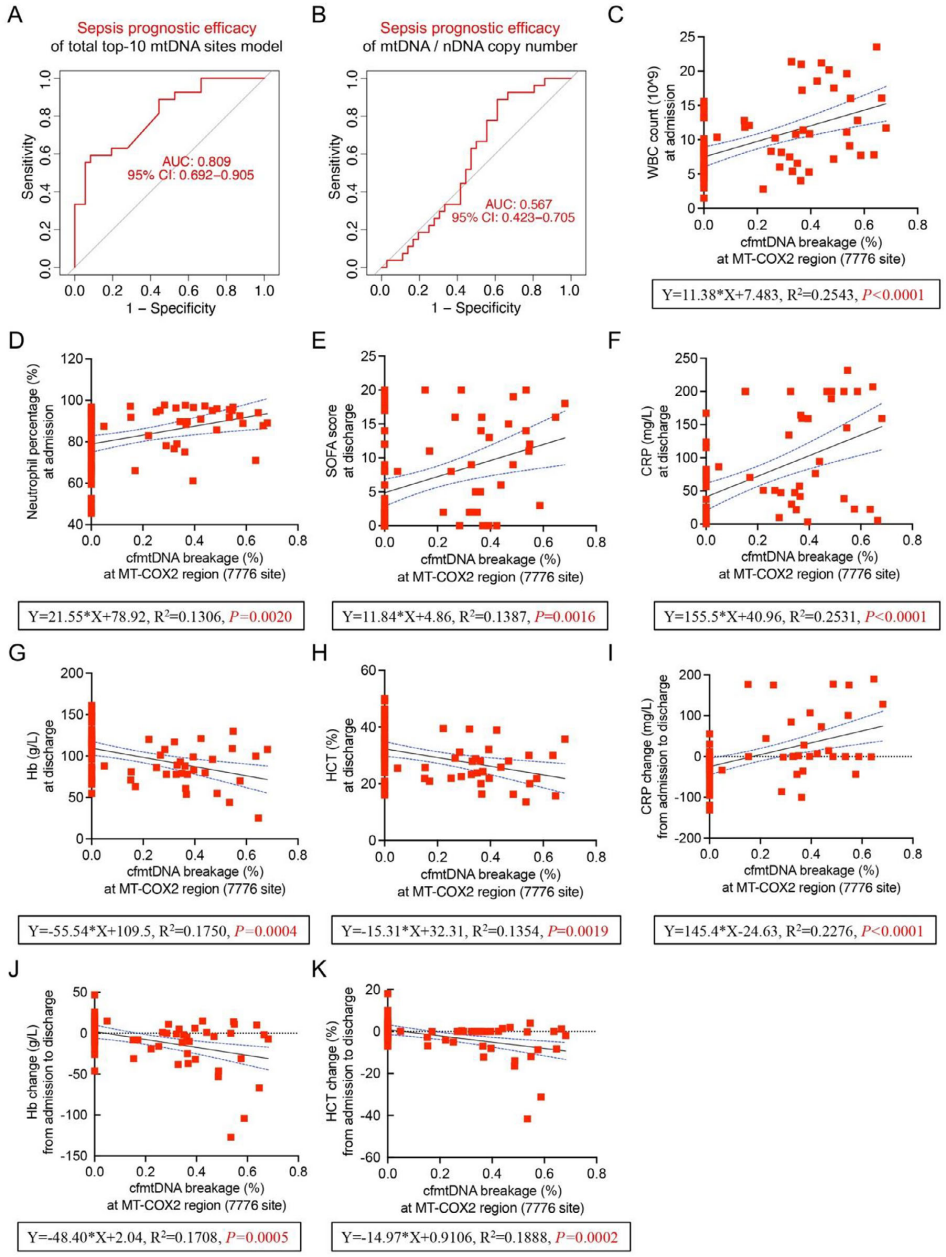
Sepsis prognostic efficacy of plasma cfmtDNA fragmentation patterns. A) Prognostic efficacy of the total top‐10 cfmtDNA sites model for sepsis. B) Prognostic efficacy of the mtDNA/nDNA copy number ratio. C,D) Correlation analysis between cfmtDNA breakage frequency at admission indicators: MT‐COX2 region and WBC count (C) and neutrophil proportion (D). E–H) Correlation analysis between cfmtDNA breakage frequency in the MT‐COX2 region and discharge indicators: SOFA score (E), CRP (F), Hb (G), and HCT (H). I–K) Correlation analysis between cfmtDNA breakage frequency in the MT‐COX2 region and changes in indicators from admission to discharge: CRP (I), Hb (J) and HCT (K). The discharge timepoint refers to the final outcome timepoint, which includes both hospital discharge and in‐hospital death. *p* < 0.05 was considered statistically significant.

ROC curve analysis revealed an AUC of 0.809 when the top‐10 cfmtDNA breakage sites were compiled in a composite model for prognostic assessment in patients with sepsis (**Figure** **4**A), significantly outperforming the predictive capacity of mtDNA copy number for poor prognosis (AUC = 0.567; Figure 4B). This suggests that the severity of high‐frequency breakage at specific cfmtDNA sites in the plasma at the time of admission can serve as an indicator of sepsis prognosis.

To further investigate the relationship between cfmtDNA breakage characteristics and the clinical indicators of sepsis, we conducted a correlation analysis focusing on the percentage of breakage at site 7776 in the COX2 region. The breakage percentage at site 7776 in the COX2 region of plasma cfmtDNA at admission positively correlated with white blood cell count (P < 0.0001, Figure 4C) and neutrophil proportion (P = 0.0020, Figure 4D) at admission. Additionally, it positively correlated with SOFA scores (P = 0.0016, Figure 4E) and CRP levels (P < 0.0001, Figure 4F) at discharge and negatively correlated with hemoglobin concentration (P = 0.0020, Figure 4G) and hematocrit (HCT; P = 0.0016, Figure 4H) at discharge. Moreover, the breakage percentage at site 7776 positively correlated with changes in CRP during hospitalization (P < 0.0001, Figure 4I) and negatively correlated with changes in hemoglobin (P = 0.0005, Figure 4J) and HCT (P = 0.0002, Figure 4K) during hospitalization. Thus, the severity of high‐frequency breakages in the COX2 region of plasma cfmtDNA at admission may be an early warning indicator of poor prognosis in patients with sepsis.

### 2.4 Reduced Protein Binding Levels of Plasma cfmtDNA in Sepsis Expose Specific Sites to Cleavage by Bacteria‐Released Restriction Endonucleases

**Figure 5:**
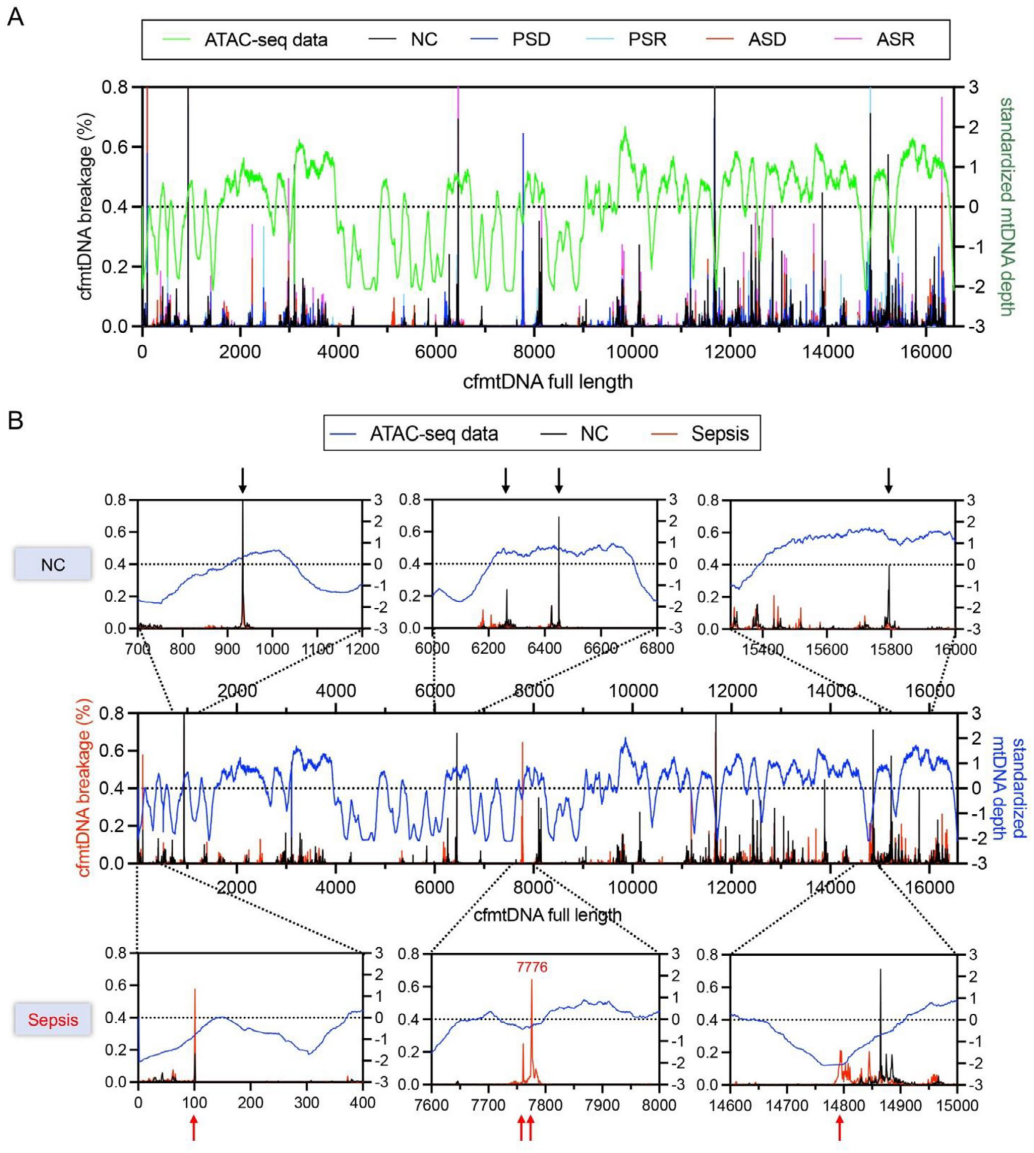
ATAC sequencing reflects protein binding levels across the full‐length cfmtDNA sequence. A) Protein binding levels of cfmtDNA, as reflected by ATAC sequencing‐based standardized mtDNA depth and cfmtDNA breakage frequency across the full‐length cfmtDNA in different sepsis subgroups. B) Representative mtDNA sites demonstrate the sequential relationship between mtDNA breakage and the weakened protein binding, leading to full mtDNA exposure.

To investigate the potential mechanisms underlying cfmtDNA breakage in the plasma of patients with sepsis, ATAC sequencing was performed to assess protein binding levels across the full‐length cfmtDNA sequences. Potential breakage sites in cfmtDNA were predominantly located in regions with lower protein binding in healthy individuals and patients with sepsis (**Figure** **5**A). This suggests that stronger protein binding reduces the likelihood of breakage at these sites. Further analysis indicated that under normal conditions, cfmtDNA breakage sites were primarily located at the terminal phase of increased standardized mtDNA depth or peak regions (Figure 5B), implying that breakage typically occurred only in areas with minimal protein binding and fully exposed mtDNA. However, in sepsis, breakage sites (e.g., COX2‐7776) were predominantly found in the initial phase of increased standardized mtDNA depth (Figure 5B), suggesting that cfmtDNA breakage can occur as soon as protein binding begins to decrease before the full exposure of the mtDNA fragment. This may explain why cfmtDNA breakage patterns provide an earlier warning of sepsis onset and prognosis than an increase in the mtDNA copy number.

**Figure 6:**
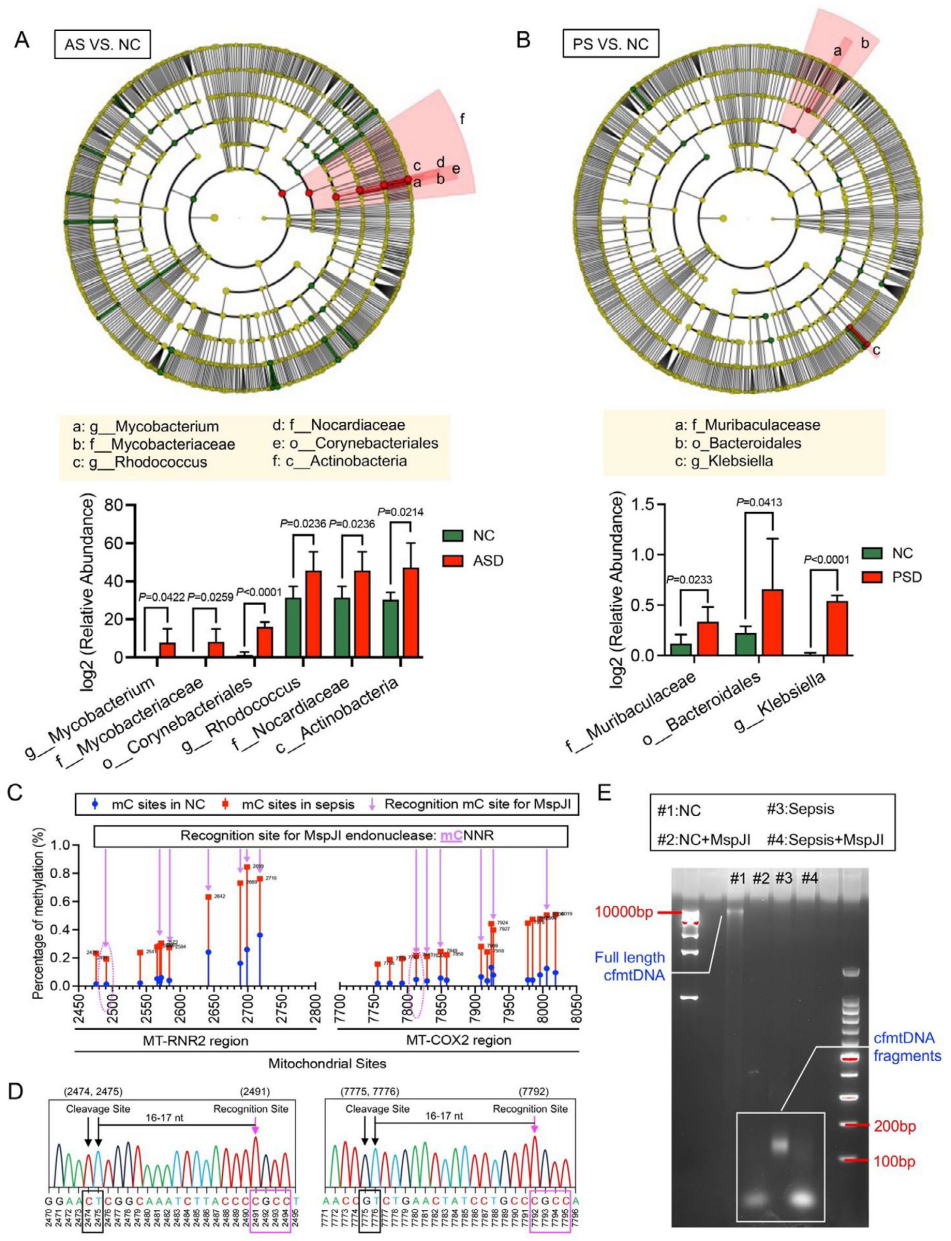
Methylation and fragmentation of plasma cfmtDNA in sepsis and its association with bacteria‐released restriction endonucleases. A,B) Changes in plasma microbiota in different sepsis groups.16S rRNA gene sequencing results reveal significant differences in the bacterial composition of plasma from patients with sepsis caused by abdominal infection (AS) (A) or pulmonary infection (PS) (B). C) Methylation of cfmtDNA and its recognition by MspJI in sepsis plasma. D) Electropherogram traces illustrating high‐frequency cfmtDNA breakage sites recognized by MspJI. E) Nucleic acid electrophoresis demonstrating fragmented cfmtDNA in sepsis plasma.

We also examined changes in plasma microbiota among patients with different types of sepsis. The 16s rRNA gene sequencing results revealed significant differences in the bacterial types in the plasma following sepsis, whether caused by abdominal infection (AS) or pulmonary infection (PS). Specifically, the relative abundances of Mycobacterium, Rhodococcus, and Klebsiella were significantly elevated in the plasma of these patients (**Figure** **6**A,B). These bacteria release restriction endonucleases, such as MspJI, into the bloodstream. MspJI recognizes cytosine residues modified at the C‐5 position, particularly 5‐methylcytosine (5mC) and 5‐hydroxymethylcytosine (5hmC), and cleaves 16–17 bases downstream of the recognition site.^[^ ^14^ ^]^ Methylation analysis of cfmtDNA revealed that several CNNR sites in plasma cfmtDNA from patients with sepsis were methylated (mCNNR). Most of these sites were recognized by the bacterial restriction endonuclease MspJI (Figure 6C). Representative electropherogram traces demonstrated that the methylated cfmtDNA sites recognized by MspJI generated cleavage sites 16–17 bases downstream, corresponding to the high‐frequency breakage sites identified in the plasma cfmtDNA of patients with sepsis, such as 2474, 2475, 7775, and 7776 (Figure 6D). These results suggest that methylation‐exposed sites of plasma cfmtDNA in sepsis are recognized and cleaved by bacteria‐released restriction endonucleases. Nucleic acid electrophoresis further confirmed that the fragmentation of plasma cfmtDNA in patients with sepsis was likely associated with the cleavage effects of bacterial restriction endonucleases, such as MspJI (Figure 6E).

Cumulatively, these results indicate that reduced protein binding levels in plasma cfmtDNA during sepsis expose specific sites, making them more susceptible to cleavage. Hence, the increase in restriction endonucleases released by bacterial infections may directly cause cfmtDNA fragmentation.

## 3 Discussion

Mitochondrial DNA (mtDNA) has increasingly become a critical focus in biomedical research. Traditionally, researchers have focused on measuring mtDNA copy numbers to evaluate cellular health, mitochondrial function, and their associations with various diseases. However, with the advancement of molecular biology techniques and a deeper understanding of mitochondrial function, it has become clear that relying solely on mtDNA copy numbers is insufficient to meet the needs of contemporary research. While mtDNA copy number analysis provides an overall estimate of the mitochondrial genome quantity by quantifying relatively stable and easily amplifiable genes, such as MT‐ND1, MT‐ND6, CYB, COX1, and COX3, this approach is limited in its ability to capture complex, dynamic changes in mitochondrial function. This is particularly problematic in the pathological study of complex diseases, where critical information may be missed.^[^ ^15^ ^]^ Although previous research has indicated that increased plasma mtDNA copy numbers are characteristic of acute severe injuries, such as sepsis,^[^ ^16^ ^]^ the sensitivity of this measure was found to be less than ideal in the current study. Significant increases in plasma mtDNA copy number were observed only in the PSD group, with no significant differences detected in the PSR, ASD, and ASR groups. This suggests that relying solely on the mtDNA copy number to assess sepsis severity is insufficiently sensitive and may only provide early clinical warnings in cases of severe abdominal infections leading to sepsis.

In this study, we applied mitochondrial DNA fragmentomics using single‐primer amplification and library construction technology to achieve early diagnosis and prognosis assessment of clinical sepsis. This method enables the single‐molecule detection of cell‐free nucleic acids through a three‐step reaction, significantly enhancing detection sensitivity and specificity. The innovations of this strategy include using a specially designed single biphasic primer for highly specific multiplex linear amplification, successfully overcoming the limitations of traditional PCR techniques for detecting short nucleic acid fragments.^[^ ^13^ ^]^ We also introduced specially modified dNTP molecules for efficient labeling combined with a nucleic acid affinity purification system, markedly improving the recovery rate of short nucleic acid fragments. Subsequently, a single‐stranded DNA ligase was used to construct a highly specific library of molecules to ensure detection accuracy and reliability. Finally, by applying unique molecular identifier (UMI) deduplication and single‐strand selective amplification noise reduction strategies, we effectively suppressed noise in the detection process, significantly increasing the detection efficiency of rare, short nucleic acid fragments by five to ten times compared with existing techniques. This approach highlights the potential application value of plasma cfmtDNA breakage characteristics in precision medicine and the early diagnosis and prognosis assessment of sepsis. The current turnaround time for cf‐mtDNA breakage pattern analysis is ≈6–8 h, including sample processing, library preparation, and sequencing. Future improvements, such as the automation of sample preparation and the adoption of rapid sequencing platforms, could further reduce the detection time and improve the feasibility of applying this strategy in clinical practice. In addition, the cf‐mtDNA breakage pattern analysis may be used in combination with conventional biomarkers such as SOFA, CRP, and PCT to improve the clinical utility of sepsis diagnosis and prognosis. While these traditional markers are valuable in identifying established sepsis and gauging disease severity, they primarily reflect systemic inflammation and organ dysfunction that have already occurred. In contrast, cf‐mtDNA breakage patterns may serve as much earlier indicators of mitochondrial injury, which precedes overt tissue damage or clinical deterioration. This temporal advantage suggests that cf‐mtDNA breakage analysis could offer a more sensitive window for early risk stratification. When used alongside SOFA, CRP, and PCT, this approach could enhance diagnostic accuracy, identify high‐risk patients earlier, and guide clinical intervention with greater precision.

Mitochondrial DNA fragmentomics has recently emerged as a vital tool for elucidating changes in mtDNA during pathological processes and for exploring specific disease biomarkers.^[^ ^13^ ^]^ Traditionally, mtDNA copy number analysis involves qPCR to amplify mitochondrial and nuclear genes using specific primers, with copy numbers inferred from fluorescence intensity determined using standard curves or relative comparisons.^[^ ^12^ ^]^ In contrast, mitochondrial DNA fragmentomics uses high‐throughput sequencing (next‐generation sequencing) combined with PCR amplification. This approach quantifies different fragments while facilitating an in‐depth analysis of fragment lengths, distribution, mtDNA breakage patterns, breakpoints, rearrangements, and mutation locations and frequencies.^[^ ^13^ ^]^ The depth and complexity of this analysis far exceed those of traditional mtDNA copy number analyses, providing crucial insights into the roles of mtDNA in stress responses, aging, cancer, neurodegenerative diseases, and other conditions. Kim et al. analyzed cfmtDNA fragment size information in tumor patients, effectively distinguishing tumor‐associated DNA from nuclear DNA, significantly improving the accuracy of detecting tumor‐derived mutations, and providing reliable biomarkers for early tumor diagnosis.^[^ ^17^ ^]^ Similarly, a study by Qi et al. in patients with neurodegenerative diseases, such as Alzheimer's disease, revealed that cfmtDNA fragment length and breakage site characteristics reflect mitochondrial dysfunction, aiding in early diagnosis and potentially serving as crucial tools for assessing disease severity and monitoring treatment responses.^[^ ^18^ ^]^ In the current study, specific breakages in the RNR2 and COX2 regions of plasma cfmtDNA were identified as potential early diagnostic markers of sepsis. Moreover, the degree of high‐frequency breakage in MT‐COX2 at the time of patient admission may represent an early warning indicator of poor prognosis in sepsis. This discovery provides new molecular markers for early sepsis diagnosis and establishes a robust foundation for sepsis treatment strategies based on mitochondrial quality monitoring, addressing the lag in clinical indicators of organ damage and offering significant clinical value. The shift from mtDNA copy number analysis to mitochondrial DNA fragmentomics is a natural extension of technological advancement and a necessary step toward advancing mitochondrial research to deeper, more precise levels.^[^ ^19^ ^]^

mtDNA was once considered exempt from epigenetic regulation due to its unique circular structure and replication mechanism distinct from nuclear DNA. However, recent studies have challenged this view by revealing that mtDNA undergoes methylation under certain conditions that may play significant roles in various pathological states.^[^ ^20^, ^21^ ^]^ In 2011, researchers discovered that DNMT1 contains a mitochondrial targeting sequence (MTS), enabling it to enter the mitochondria and perform methylation transfer functions.^[^ ^21^ ^]^ Subsequent studies have identified other DNA methylation‐related enzymes within the mitochondria, such as DNMT3A, DNMT3B, and TET1/2/3.^[^ ^20^ ^]^ Indeed, mitochondrial DNA methylation reportedly exhibits strand specificity, with significantly higher methylation levels on the light strand (L‐strand) than the heavy strand (H‐strand), primarily at non‐CpG asymmetric sites. The methylation of mitochondrial genes and gene boundary regions may be critical in regulating mitochondrial transcription.^[^ ^22^ ^]^ The clinical significance of mtDNA methylation modifications has been widely reported. For instance, exposure to polycyclic aromatic hydrocarbon pollutants is significantly correlated with platelet mtDNA methylation levels.^[^ ^23^ ^]^ Moreover, mitochondrial DNA methylation is reportedly disrupted in neurodegenerative diseases and may serve as a potential biomarker for oxidative stress, inflammation, and pro‐oxidative environmental exposure.^[^ ^24^ ^]^ In the current study, we discovered that the promoter regions of cfmtDNA contain multiple potentially methylated CpG islands. Furthermore, the methylation levels of cfmtDNA in the plasma of patients with sepsis were significantly elevated and closely associated with specific breakage sites in the RNR2 and COX2 regions. However, the mechanisms linking mtDNA breakage sites to methylation require further investigation.

Proteins bound to mtDNA are essential for maintaining DNA integrity. Transcription factors, histone‐like proteins, and other DNA‐binding proteins within the mitochondria bind to specific sites on mtDNA, protecting it from external damage. In particular, under conditions of cellular homeostasis, these proteins effectively shield mtDNA, preventing nuclease attacks and DNA breakage. Liu et al. revealed that under stress conditions, the binding of mtDNA to the autophagy receptor TFAM limits inflammatory responses, helping to maintain cellular stability.^[^ ^25^ ^]^ Moreover, mtDNA binds to TLR9 during myocardial ischemia‐reperfusion injury, activating the NLRP3 inflammasome and cGAS–STING signaling pathway and regulating the inflammatory response.^[^ ^26^ ^]^ In our study, in sepsis, the binding capacity of mtDNA to protective proteins was significantly reduced, exposing previously shielded sites. These exposed sites, including the five consecutive sites in the RNR2 region (residues 2474, 2475, 2476, 2477, and 2478) and five relatively consecutive sites in the COX2 region (residues 7761, 7775, 7776, 7777, and 7783), become more susceptible to recognition and cleavage by restriction endonucleases. This may further exacerbate mitochondrial dysfunction, leading to increased cell death and intensified inflammatory responses. This newly defined relationship between reduced mtDNA protein binding and restriction endonuclease‐mediated mtDNA breakage provides a new direction for developing precise diagnostic and therapeutic strategies based on mtDNA breakage patterns, particularly for the early detection and intervention of inflammatory diseases and the regulation of inflammatory responses. It also offers new possibilities for identifying molecular targets for related treatments.

Bacteria may release restriction endonucleases when infecting a host, which recognize and cleave the host DNA, including mtDNA, causing damage to the host cells and triggering further inflammatory responses.^[^ ^9^ ^]^ Although the specific mechanisms of restriction endonucleases in the human body are under investigation, evidence suggests that bacteria release these enzymes during certain infectious diseases to disrupt host cells. For example, in intestinal infections, gram‐negative bacteria, such as the Enterobacteriaceae family, secrete restriction endonucleases that target and cleave host cell DNA, leading to tissue damage.^[^ ^27^ ^]^ Similarly, in chronic urinary tract infections, Escherichia coli and Klebsiella can release restriction endonucleases, attack the host epithelial or immune cells, and cause sustained tissue damage.^[^ ^9^ ^]^ Bacterial exotoxins and endotoxins induce the release of restricted endonucleases. These enzymes directly cleave host mtDNA, causing mtDNA fragments to enter the cytoplasm or bloodstream in damage‐associated molecular patterns (DAMPs).^[^ ^28^ ^]^ Once recognized by the immune system, these DAMPs activate the innate immune response, exacerbating the inflammatory reaction and worsening the pathological state caused by the infection. These mechanisms reveal the potential pathological role of restriction endonucleases in infectious diseases and their crucial role in disease progression.

Bacteria‐released restriction endonucleases recognize specific sequences within mtDNA, such as unmethylated CpG islands or other methylation modification sites, and selectively cleave these sites. For example, MspJI can recognize 5‐methylcytosine‐modified DNA sequences and cleave nearby.^[^ ^28^ ^]^ This partially explains the phenomenon of high‐frequency mtDNA breakage under pathological conditions. We found that the destructive effect of restriction endonucleases released by bacteria on mtDNA was particularly significant in severe infections such as sepsis. Moreover, the high‐frequency breakage sites in the mtDNA closely matched the methylation sites recognized by MspJI, indicating that the activity of this enzyme may be a direct cause of mtDNA cleavage. Future research should elucidate how bacterial restriction endonucleases recognize and cleave mtDNA to better understand the interactions between bacterial infections and inflammatory responses.

This study had the following limitations: 1) As the first to apply mitochondrial DNA fragmentomics technology for the early diagnosis and prognostic assessment of sepsis, this research only conducted a representative analysis of 10 sites within the COX2 and RNR2 regions of mtDNA, which are absolutely conserved in healthy individuals but exhibit high‐frequency breakage post‐sepsis. Whether other sites hold similar clinical diagnostic value requires further investigation. 2) The relationship between the weakened binding ability of mtDNA proteins and the restriction enzyme‐mediated mtDNA breakage still requires in‐depth mechanistic exploration. 3) Due to the complexity of bacterial infections in sepsis, the damaging effect of bacterial restriction enzymes on cfmtDNA needs further study. In this study, 16S rRNA sequencing was performed on plasma samples to verify the presence of circulating microbial DNA and support the systemic infectious state. However, the comprehensive characterization of microbial communities at the primary infection sites (e.g., lungs or abdominal cavity) was beyond the scope of this study and should be addressed in future investigations. 4) In future research, we plan to conduct large‐scale, multi‐center prospective studies to overcome the limitation of sample size. We will also include critically ill patients without sepsis for comparison, in order to provide stronger evidence on whether the observed cf‐mtDNA breakage patterns are disease‐specific to sepsis.

## 4 Conclusion

This study is the first to apply mitochondrial DNA fragmentomics technology for the early diagnosis and prognostic assessment of sepsis, highlighting the clinical potential of cfmtDNA breakage patterns. Specific breakages in the RNR2 and COX2 regions of plasma cfmtDNA provide greater sensitivity and specificity for early diagnosis of sepsis than traditional cfmtDNA copy number analysis. Notably, high‐frequency breakage in the COX2 region is strongly associated with poor prognosis, positioning it as a potential early warning indicator. Further analysis revealed that in patients with sepsis, decreased protein‐binding levels in plasma cfmtDNA exposed sites vulnerable to cleavage by bacteria‐released restriction endonucleases, leading to high‐frequency breakage. These insights offer new directions for advancing early diagnosis and prognosis assessment and developing therapeutic targets for sepsis.

## 5 Experimental Section

### Patients Recruitment and Plasma Sample Collection

From September 2022 to September 2023, 63 patients with suspected sepsis admitted within 24 h of hospital admission and 10 non‐sepsis control patients were recruited from the Second Affiliated Hospital of Chongqing Medical University, Xinqiao Hospital of the Army Medical University, and Jinling Hospital of Nanjing University, China. Sepsis was defined according to the Sepsis‐3.0 criteria as life‐threatening organ dysfunction resulting from a dysregulated host response to infection. Informed consent was obtained from all eligible patients. If the patient was unable to provide consent due to medical conditions, the next of kin was asked to provide written informed consent, followed by confirmatory consent from the patient when conditions allowed. The institutional Clinical Research Ethics Committee approved the patient recruitment and data collection protocols (Nos. 2022XGIIT02 and 2022–190). This study was conducted in compliance with the principles of the Declaration of Helsinki.

Blood samples were collected on the first day of hospital admission. To minimize the confounding effects of antibiotics, blood specimens were collected as early as possible after the suspicion of sepsis or after ICU admission for a septic episode. For each sample, 3 mL of blood was obtained by venous puncture using aseptic technique and was stored in a K2EDTA tube at 4 °C. The plasma was separated within 2 h of blood collection. Briefly, whole blood was centrifuged at 1200 × g and 4 °C for 10 min; the supernatant was collected and further centrifuged at 12 000 × g and 4 °C for 10 min. The separated plasma was stored at −80 °C until further processing.

### Extraction and Quantification of Cell‐Free DNA (cfDNA)

Plasma samples were thawed at room temperature before cfDNA extraction using a HiPure Circulating DNA Midi Kit C (Magen, Guangzhou, China) per the manufacturer's protocol. The integrity of the extracted DNA was evaluated by agarose gel electrophoresis or using an Agilent 4200 TapeStation system (Agilent Ltd., Waldbronn, Germany) to assess potential degradation and RNA contamination. DNA purity was measured based on the OD260/280 ratio using a NanoDrop One spectrophotometer (Thermo Fisher Scientific, Waltham, MA, USA). Finally, the concentration of extracted DNA was quantified using a Qubit 3.0 Fluorometer (Thermo Fisher Scientific).

### Cell‐Free Mitochondrial DNA (cfmtDNA) Library Construction and Data Processing

Primers were designed based on the human mitochondrial genome (version: GRCh38.p13) with an average fragment length of 130 bp. Plasma mitochondrial DNA libraries were constructed using an OPERA Jupitor DNA Universal Library Preparation Kit (APG‐62001; Apogenomics, Shanghai, China). Sequencing was performed with the Illumina NovaSeq platform at Sinotech Genomics Co., Ltd. (Shanghai, China).

Sequencing reads were initially processed using Cutadapt to remove adapter sequences. After trimming, the reads were sorted into sub‐FASTQ files according to their primer sequences. The sub‐FASTQ files were aligned to the reference genome (GRCh38.p13) using the BWA (v0.7.13) MEM algorithm. On‐target analysis was performed with SAMtools, and deduplication was performed using Fgbio to generate BAM files, providing mitochondrial gene alignment and quantification.

The BAM files were visualized using the Integrative Genomics Viewer (IGV) to examine the depth at various breakpoints. Breakpoint frequencies at different loci were illustrated with PRISM. Breakpoint analysis was conducted based on the depth at each target region locus as follows:

1. The number of molecules at breakpoint n was calculated as the deduplicated depth at locus n minus the deduplicated depth at locus n+1.
2. The frequency of breakpoint n was determined by dividing the number of molecules at breakpoint n by the total number of deduplicated molecules, multiplied by 100.

### Quantitation of Cell‐Free Nuclear DNA (cfnDNA) and Mitochondrial DNA (cfmtDNA) in Plasma

Quantification of cfnDNA and cfmtDNA in plasma was performed using SYBR Green‐based real‐time quantitative PCR (RT‐qPCR), as previously described.^[^ ^29^ ^]^ For human nuclear genomic targets, the reference genes were telomerase reverse transcriptase (TERT) and glyceraldehyde‐3‐phosphate dehydrogenase (GAPDH), whereas for human mitochondrial genomic targets, mitochondrial‐encoded NADH dehydrogenase 1 (MT‐ND1) and mitochondrial‐encoded NADH dehydrogenase 6 (MT‐ND6) served as the internal references. Real‐time qPCR assays for these gene targets were designed using Primer3 software:^[^ ^15^ ^]^

GAPDH (Forward): 5′‐GTATTCCCCCAGGTTTACATGTTC‐3′,
(Reverse): 5′‐ACTCACTCCTGGAAGATGGTGAT‐3′;
TERT (Forward): 5′‐CAATGCCTCACATAAATGCTACC‐3′,
(Reverse): 5′‐AGTGCAAAGCTTCTGTCTCCTTCT‐3′;
MT‐ND1 (Forward): 5′‐CCCTAAAACCCGCCACATCT‐3′,
(Reverse): 5′‐GAGCGATGGTGAGAGCTAAGGT‐3′;
MT‐ND6 (Forward): 5′‐CCAATCCTACCTCCATCGCT‐3′,
(Reverse): 5′‐GAGTATCCTGAGGCATGGGG‐3′.

### ELISA Detection of MT‐RNR2 and MT‐COX2

MT‐RNR2 and MT‐COX2 levels were evaluated with the respective ELISA kits: Human Putative Humanin Peptide (MT‐RNR2) ELISA Kit (CSB‐EL015084HU, Cusabio, Wuhan, China) and Human/Mouse Total COX‐2 DuoSet IC ELISA Kit (DYC4198‐5, Bio‐Techne, Abingdon, UK), according to the manufacturers’ instructions. The optical density (OD) was measured at 450 nm with the wavelength correction set at 540 or 570 nm.

### ATAC Sequencing and Data Processing

The nuclear pellet was resuspended in a transposase reaction mix of 25 µL of 2 × TD buffer, 2.5 µL of transposase (Illumina), and 22.5 µL of nuclease‐free water. Transposition was performed at 37 °C for 30 min. Subsequently, the samples were purified using a Qiagen Minelute kit. Library fragments were amplified using NEBNext PCR master mix and custom Nextera PCR primers under the following conditions: initial extension at 72 °C for 5 min, denaturation at 98 °C for 30 s, followed by 12 cycles of 98 °C for 10 s, 63 °C for 30 s, and 72 °C for 1 min, with a final extension at 72 °C for 5 min. The amplified libraries were purified using a Qiagen PCR Cleanup Kit.

Raw sequencing reads were processed using Trimmomatic to remove adapters and short (<35 bp), low‐quality reads with the assistance of Sinotech Genomics Co., Ltd. (Shanghai, China). Quality control was performed with FastQC software.^[^ ^30^ ^]^ High‐quality reads were aligned to the human reference genome (GRCh38) using Bowtie2 v2.3.4.1.^[^ ^31^ ^]^ Reads with low mapping quality (MAPQ < 30) were filtered by Samtools, and duplicate reads were removed by Picard's MarkDuplicates. Peak calling was performed with MACS2 v2.1.2^[^ ^32^ ^]^ with a q‐value threshold of 0.05. The peaks were further refined for reproducibility using the IDR framework, retaining only those with IDR values ≤ 0.05. Peak annotation was conducted by the ChIPseeker R package,^[^ ^33^ ^]^ and motif analysis was performed using the MEME suite (http://meme‐suite.org/).

### 16S rRNA Sequencing and Data Processing

Among the 63 sepsis patients included in this study, 45 had positive blood cultures. For these patients, 16S rRNA sequencing was performed on plasma samples and showed a high level of concordance with blood culture results at the genus level in 42 out of 45 cases. Plasma microbial DNA was extracted with a QIAamp DNA Stool Mini Kit (Qiagen, Germany) and quantified using a Qubit 2.0 Fluorometer. The extracted DNA was sequenced by Biotree Biotech Co., Ltd. (Shanghai, China) using an Illumina MiSeq platform with a 500‐cycle v2 kit. The V3–V4 region of the 16S rRNA gene was amplified on an ABI 2720 Thermal Cycler (Thermo Fisher Scientific) with forward (5′‐CCTACGGGNBGCASCAG‐3′) and reverse (5′‐GGACTACNVGGGTWTCTAAT‐3′) primers. PCR products were pooled, purified using Agencourt AMPure XP magnetic beads, and assessed for quality with a NanoDrop 2000 spectrophotometer. Sequencing was performed with the Illumina MiSeq platform. Raw reads were processed using FLASH for assembly, Cutadapt for primer removal, and quality filtering. Sequences were clustered at 97% similarity into operational taxonomic units (OTUs), and taxonomic classification was performed using the SILVA132 database.

### cfmtDNA Methylation Site Detection

The extracted DNA was evaluated for degradation and RNA contamination by agarose gel electrophoresis and an Agilent 4200 TapeStation. DNA purity was assessed by measuring the OD260/280 ratio using a Nanodrop One spectrophotometer (Thermo Fisher Scientific), and DNA concentration was quantified using a Qubit 3.0 fluorometer (Thermo Fisher Scientific). A total of 5 to 500 ng of DNA was then subjected to bisulfite conversion using the EZ DNA Methylation‐Gold Kit (D5005, Zymo Research Corp, USA),^[^ ^34^ ^]^ following the manufacturer's instructions.

Primers targeting specific methylation sites were designed for library preparation, and libraries were constructed using the OPERA Mars Methylation DNA Library Prep Kit (APG‐62002, Apogenomics, Shanghai, China) for Illumina sequencing. Sequencing was performed on the Illumina NovaSeq 6000 platform.

After sequencing, the raw FASTQ data were subjected to quality control using Fastp and multiQC. The bisulfite‐converted reads were aligned to the reference genome using Bismark with Bowtie2 as the aligner. The resulting BAM files were evaluated for quality with Qualimap, bedtools, and SAMtools, focusing on alignment efficiency and target region capture. Genome‐wide methylation analysis was conducted using the methylKit, including identification of differentially methylated sites (DMS) and regions (DMR), followed by annotation and enrichment analysis of the identified DMRs.

### Agarose Electrophoresis

Agarose gel (2%) was prepared by dissolving 2 g of RNase‐free agarose in 100 mL of TAE buffer and pouring it into cast trays. For Northern blotting, gels were prepared using 1 × NorthernMax‐Gly buffer. RNA samples were denatured by mixing with glyoxal‐containing sample buffer at a 1:1 ratio, heating at 50 °C for 30 min, and cooling on ice for at least 3 min. Denatured RNA was loaded into the wells and subjected to electrophoresis at 50 V and room temperature until the bromophenol blue dye reached the edge of the gel. Following electrophoresis, the gels were stained with SYBR‐Safe (diluted 1:10000 in 1 × NorthernMax‐Gly buffer) and imaged with a Bio‐Rad Gel Doc XR system and Image Lab 5.2 software, utilizing the “SYBR‐Safe” settings.

### Statistical Analysis

Statistical analyses were performed using SPSS (version 25.0; IBM Corp., Armonk, NY, USA) and GraphPad Prism version 8.0 (GraphPad Software, San Diego, CA, USA). Continuous variables were presented as mean ± standard deviation (SD) or median with interquartile range (IQR), depending on the data distribution as assessed using the Shapiro‐Wilk test. For group comparisons, an independent t‐test or Mann‐Whitney U‐test was applied for two groups based on normality. A one‐way analysis of variance ANOVA or Kruskal–Wallis test was used to compare more than two groups. Post‐hoc analyses were performed using Tukey's Honestly Significant Difference (HSD) test for ANOVA and Dunn's test for the Kruskal–Wallis test. Categorical variables were analyzed using the chi‐squared test or Fisher's exact test, as appropriate. Correlations between variables were assessed using Pearson's correlation coefficient for normally distributed data and Spearman's rank correlation coefficient for nonparametric data. Receiver operating characteristic (ROC) curve analysis was employed to evaluate the diagnostic performance of biomarkers. The area under the curve (AUC) was calculated, and the optimal cutoff point was determined based on the Youden index. All statistical tests were two‐tailed, and a P‐value < 0.05 was considered statistically significant.

## Conflict of Interest

The authors declare no conflict of interest.

## Author Contributions

C.D. performed conceptualization. H.L., X.T., B.C., S.H., and Z.C. performed data curation. J.H. and W.S. performed formal analysis. C.D., H.H., and Z.L. performed funding acquisition. S.H. performed investigation. H.L., J.H., and W.S. performed methodology. C.D., R.M., and L.L. performed supervision. X.Z. and R.S. performed visualization. C.D. wrote the original draft. C.D. and L.L. wrote, review and edited the final manuscript.

## Supporting information



Supporting Information

## Data Availability

The data that support the findings of this study are available from the corresponding author upon reasonable request.
